# Local Evolution of Seed Flotation in Arabidopsis

**DOI:** 10.1371/journal.pgen.1004221

**Published:** 2014-03-13

**Authors:** Susana Saez-Aguayo, Corinne Rondeau-Mouro, Audrey Macquet, Ilkka Kronholm, Marie-Christine Ralet, Adeline Berger, Christine Sallé, Damien Poulain, Fabienne Granier, Lucy Botran, Olivier Loudet, Juliette de Meaux, Annie Marion-Poll, Helen M. North

**Affiliations:** 1 INRA, Institut Jean-Pierre Bourgin, UMR1318, Saclay Plant Sciences, Versailles, France; 2 AgroParisTech, Institut Jean-Pierre Bourgin, UMR1318, Saclay Plant Sciences, Versailles, France; 3 INRA, UR 1268 Biopolymères Interactions Assemblages, INRA, Nantes, France; 4 Irstea, UR TERE, CS 64427, Rennes, France; 5 Department of Genetics and Plant Breeding, Max Planck Institute for Plant Breeding Research, Cologne, Germany; 6 Institute of Evolution and Biodiversity, University of Münster, Münster, Germany; Harvard University, United States of America

## Abstract

Arabidopsis seeds rapidly release hydrophilic polysaccharides from the seed coat on imbibition. These form a heavy mucilage layer around the seed that makes it sink in water. Fourteen natural Arabidopsis variants from central Asia and Scandinavia were identified with seeds that have modified mucilage release and float. Four of these have a novel mucilage phenotype with almost none of the released mucilage adhering to the seed and the absence of cellulose microfibrils. Mucilage release was modified in the variants by ten independent causal mutations in four different loci. Seven distinct mutations affected one locus, coding the MUM2 β-D-galactosidase, and represent a striking example of allelic heterogeneity. The modification of mucilage release has thus evolved a number of times independently in two restricted geographical zones. All the natural mutants identified still accumulated mucilage polysaccharides in seed coat epidermal cells. Using nuclear magnetic resonance (NMR) relaxometry their production and retention was shown to reduce water mobility into internal seed tissues during imbibition, which would help to maintain seed buoyancy. Surprisingly, despite released mucilage being an excellent hydrogel it did not increase the rate of water uptake by internal seed tissues and is more likely to play a role in retaining water around the seed.

## Author Summary

Seeds of the model plant Arabidopsis release sticky mucilage on imbibition that is constituted of complex polysaccharides. In this study, we have identified and characterised natural Arabidopsis variants that do not release mucilage and found that their seeds float. The accumulation of unreleased polysaccharides in the seed coat reduced water uptake rates on imbibition and would maintain buoyancy. We subsequently identified additional floating natural variants where mucilage is released, but is not attached to the seed, apparently due to defective cellulose production. The different variants arise from at least ten independent unique mutations and were collected from two discrete geographical areas. Arabidopsis seed flotation has thus evolved several times due to modifications in mucilage release. Released mucilage was found to retain water, but did not improve imbibition of internal seed tissues, indicating a role in maintaining seeds hydrated. These findings highlight the physical and potential physiological effects of mucilage production by the seed coat.

## Introduction

Polysaccharides released from the seed coat on imbibition form a sticky, gelatinous halo called mucilage around the seed. This property, termed myxospermy, was observed in cress (*Lepidium sativum*) by Darwin [1] and is found in the model plant Arabidopsis. In addition to the *Brassicaceae*, this trait has been noted in a hundred plant families including *Solanaceae*, *Linaceae* and *Plantaginaceae* [2]. During seed development in Arabidopsis, the epidermal cells of the seed coat undergo a complex differentiation process during which mucilage polysaccharides are accumulated [3]–[5]. The resulting epidermal cells of mature Arabidopsis seeds have a distinctive morphology with reinforced radial cell walls connected to a column of secondary cell wall material at their centre, called the columella, which is surrounded by dehydrated mucilage polysaccharides under a primary cell wall. Genes involved in the differentiation of these cells and the production of mucilage have mainly been identified through mutant phenotypes [2].

In Arabidopsis, seed mucilage forms two structurally distinct layers with the pectin domain rhamnogalacturonan I (RG I) the major component of each [4], [6], [7]. The outer mucilage layer is diffuse and water-soluble. In contrast, the inner layer adheres strongly to the seed surface and requires harsh chemical treatment, or enzyme digestion, to remove it from the seed coat [4], [7]–[9]. RG I attachment to the seed coat requires cellulose, as *cesa5*, *fei2* and *sos5* mutants implicated in cellulose synthesis have reduced adherent mucilage [10]–[12]; *cesa5* is affected in a cellulose synthase catalytic subunit, *fei2* is defective in a leucine-rich receptor kinase and *sos5* carries a mutation in a fasciclin-like arabinogalactan protein with a glycophosphatidylinositol anchor. In *cesa5* null mutants some cellulose was still observed within the reduced layer of adherent mucilage, implicating other *CESA* genes in its production. Precisely how pectin and cellulose interact to form the adherent mucilage layer has still to be determined.

The ecophysiological role of mucilage production by seeds is ambiguous, diverse functions have been put forward, but none appears to be comprehensively applicable. The adhesive properties of mucilage led to proposals that it mediates long-distance seed dispersal by attachment to animals or that it prevents seed removal during soil erosion or by ants through fixation to soil particles [1], [13]–[15]. Comparison of the formation of mucilage in *Artemisia* taxa associated the trait with dry habitats, as had previously been observed in Lamiaceae [16], [17]. Nevertheless, a potential role of mucilage in modifying germination capacity [6], [18]–[20] has not been consistently observed in tests with mutants defective for mucilage release [21]–[24]. Furthermore, differences in the composition and structure of mucilage layers could reflect specific physiological roles for each [25].

Naturally occurring genetic variation provides an alternative source of mutations for functional analysis and gene cloning to that of induced mutations. In *Arabidopsis thaliana* a large number of accessions are available that have been derived from seeds harvested in the wild in a variety of geographical locations. These have generally been exploited for quantitative trait locus (QTL) mapping of important agronomic traits [26]. In a previous study we identified a naturally occurring mutation in the Shahdara accession that affects the liberation of mucilage from the seed coat [25]. The Shahdara accession is defective in the MUM2 β-D-galactosidase. This enzyme trims galactan ramifications from RG I in seed mucilage, rendering it more hydrophilic, and increasing mucilage expansion on imbibition so that the outer cell wall breaks and mucilage is released [25], [27]. Except for the mucilage extrusion defect, no other visible phenotype was reported for the Shahdara accession or other *mum2* mutants. Although the genetic basis of this phenotype was elucidated, its ecological relevance was not resolved. Seeds from the Shahdara accession had been collected in Tajikistan, near to the Shokhdara River. The collection of accessions previously screened for mucilage release defects contained mainly European accessions with few from central Asia [25].

In this study, we analysed a larger panel of Arabidopsis accessions and identified a further nine genotypes that were defective in mucilage release; these were observed to float on the surface of water, unlike seeds with a thick layer of adherent mucilage that sink. A screen for seed flotation identified four other accessions whose seeds floated despite releasing mucilage, as they had little adherent mucilage. Analysis of the causal mutations responsible for the modified mucilage release observed in the variants indicated ten independent mutations that affected a minimum of four different loci and these had evolved in populations from two geographical zones, central Asia and Scandinavia. The investigation of water uptake by seeds using nuclear magnetic resonance (NMR) relaxometry showed that non-released mucilage polysaccharides contribute to the maintenance of buoyancy, and released mucilage does not improve imbibition, contrary to previous hypotheses.

## Results

### Seed mucilage retention is observed in Arabidopsis accessions from two discrete geographic locations and arises from mutations in three loci

During an expedition to central Asia, seeds were harvested from 25 plants at a site in Tajikistan believed to correspond to that where the original Shahdara population was collected, these were termed NeoShahdara (Neo) and have been shown to be closely related to the original Shahdara individual [28]. Interestingly, descendants from just eight of the twenty-five Neo plants tested had the *mum2* ^Sha^ deletion, identified as the causal mutation for mucilage retention in the original Shahdara [25]. Individuals from two Kyrgyzstan and six Scandinavian populations were also found to be defective in mucilage release [29] (Table 1); the trait was fixed in 7 of the 9 populations. In 488 accessions examined representing the species-wide distribution of Arabidopsis, 71 exhibit the mucilage release defect [25] (Table 1). The sites of origin for these accessions were confined to central Asia and Scandinavia.

**Table 1:** List of Arabidopsis accessions analysed for seed mucilage release in this study.

Population	Geographic region	N	Number of individuals affected in mucilage release
Ale	Scandinavia	19	1
Dja	Central Asia	9	9
Eid-1	Scandinavia	5	0
Had-3	Scandinavia	5	5
Kar	Central Asia	11	0
Kon-2	Scandinavia	5	0
Kvi-2	Scandinavia	5	0
Kyr	Central Asia	8	0
Lod-2	Scandinavia	5	0
Lod-3	Scandinavia	5	0
Lom-3	Scandinavia	5	5
Neo	Central Asia	25	25
Nfro-1	Scandinavia	5	5
Röd	Scandinavia	16	0
Sk-1	Scandinavia	5	5
Sku	Scandinavia	15	8
Sus	Central Asia	7	7
Tje-1	Scandinavia	5	0
Veg-1	Scandinavia	5	0
Veg-2	Scandinavia	4	0
Vgn-1	Scandinavia	5	0
Zal	Central Asia	5	0

N, number of individuals sampled in population.

**Figure 1:**
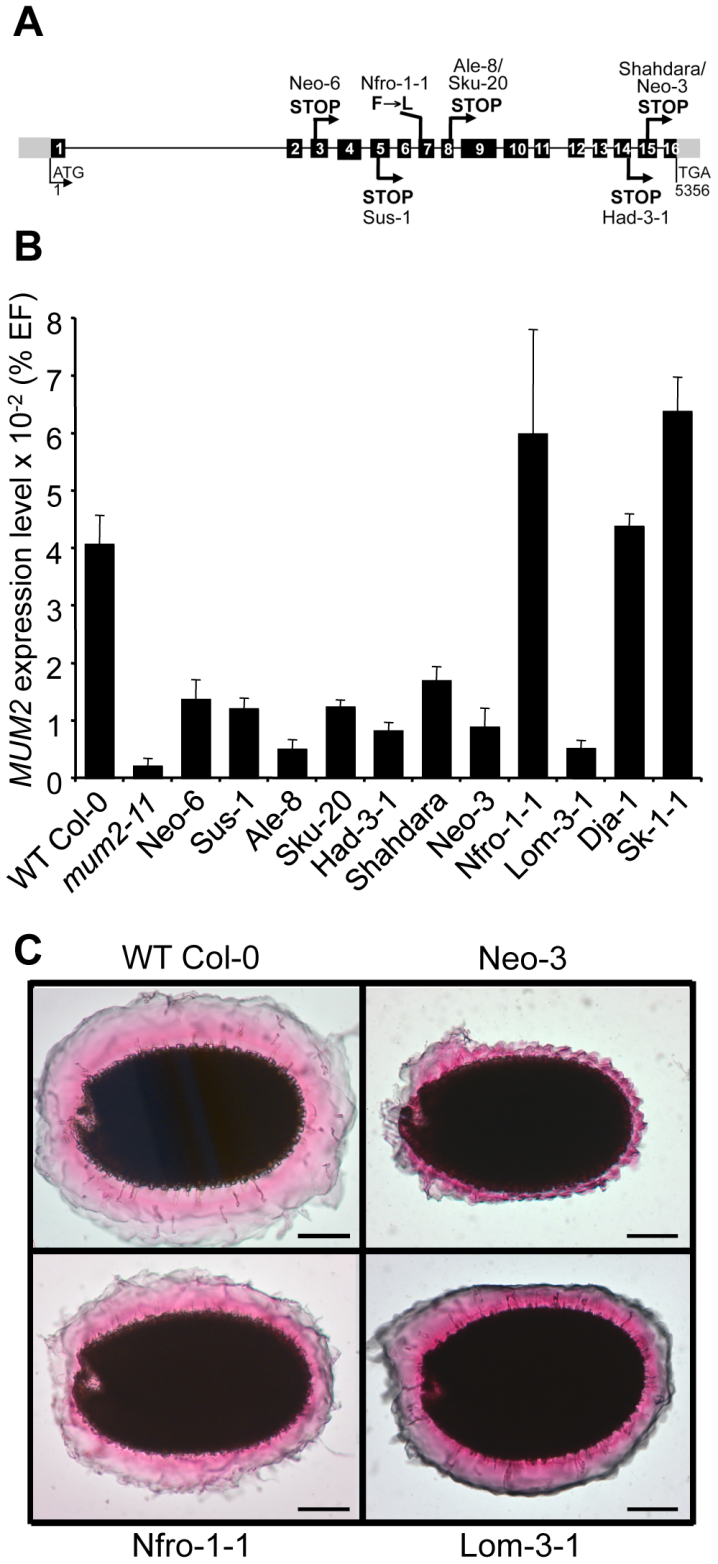
Characterization of natural *mum2* mutants. (*A*) Schematic representation of the *MUM2* gene indicating the positions of the causal mutations in the different accessions and their effect on the encoded protein in capital letters. Boxes represent exons; black shading shows coding sequence; grey shading indicates 5′ and 3′ untranslated regions. (*B*) Steady state *MUM2* mRNA levels in developing siliques of indicated accessions, 8–12 days after pollination, represented as a percentage of the constitutive *EF1α-4a* (EF) gene abundance. Error bars represent SE (n = 6). (*C*) Ruthenium red stained seeds of indicated accessions after forced mucilage release by sequential treatment with HCl and NaOH. WT, wild type.

Crosses were carried out between Shahdara and ten representative individuals from among the 70 new mutant accessions (Table S1). For Neo, a representative carrying the *mum2* ^Sha^ deletion, Neo-3, and one that did not, Neo-6, were used. Dja-1 and Sk-1-1 complemented the Shahdara *mum2* mutant phenotype. Furthermore, reciprocal crosses between Dja-1 and Sk-1-1 showed that their mutations were not allelic. The gene affecting mucilage release in Dja-1 has been identified and codes the pectin methylesterase inhibitor PMEI6 [29]. A combination of mapping and whole-genome sequencing identified a point mutation in At3g50990 in the Sk-1-1 accession; this gene codes PEROXIDASE36 (PER36) and the mutation causes the conversion of Tyr-262 to a stop codon. PER36 has recently been shown to be required for mucilage release [30]. All the remaining accessions failed to complement the Shahdara *mum2* mutant allele. *MUM2* gene polymorphisms were determined for the accessions affected in mucilage release and five causal mutations, distinct from the original Shahdara mutation, were identified in six of the accessions, four of these introduced premature stop codons (Figure 1A, Table S2). Segregation for mucilage release or non-release in the Ale and Sku populations always correlated with the absence or presence of the causal mutation, respectively.

For the Lom3-1 accession no causal mutation was identified in *MUM2*, despite genetic non-complementation demonstrating it to be a mutant allele (Table S2) and no mutation was identified in 208 base pairs (bp) upstream of the ATG or 192 bp downstream of TGA. Analysis of *MUM2* expression, by quantitative RT-PCR (qRT-PCR), confirmed that Lom3-1 was affected in *MUM2*, as RNA steady-state levels were extremely low, similar to those of the *mum2-11* knockout mutant (Figure 1B). Interestingly, *MUM2* transcript abundance was markedly reduced in all the accessions where mutation introduced a premature stop codon (Figure 1B). As these stop codons should only directly affect translation, the observed reduction in transcript levels indicates the possible intervention of nonsense mediated mRNA mechanisms to degrade aberrant transcripts [31]. As expected, *MUM2* expression levels were similar to those observed for wild-type Col-0 for the accessions Nfro1-1, Dja-1 and Sk-1-1 (Figure 1B). The latter two are not *mum2* mutants and Nfro1-1 contains an amino-acid substitution in MUM2. The natural mucilage release mutants are therefore the result of 9 independent mutation events, with 7 in the same gene *MUM2*.

The identification of *MUM2* loss-of-function alleles in two specific geographic regions could result from local selection pressures. We examined patterns of differentiation between local populations within geographical regions, using previously described patterns of neutral variation [32] (Protocol S1). Patterns of population differentiation at either the *MUM2* locus or for mucilage release did not deviate from neutral expectations (Table S3). Although the repeated evolution of independent loss-of-function mutations in central Asia and Norway remains intriguing, there is no evidence for local adaptation for mucilage retention.

### Impairment of adherent mucilage maturation by natural mutations

Forced mucilage release by breaking the outer cell wall of seed coat epidermal cells with acid and alkali has previously shown that *mum2-11* seeds form a thinner layer of adherent mucilage; branched RG I does not expand to the same degree as unbranched RG I when hydrated (Figure S1A and S1B) [11]. In accessions containing a premature stop codon, adherent mucilage was observed as a thin layer, like that of *mum2-11* seeds, coherent with reduced *MUM2* transcript abundance (Figure 1C and Figure S1C to S1H). The adherent mucilage was reduced less for Nfro1-1 and Lom-3-1 consistent with the production of normal amounts of a hypofunctional enzyme or a reduced amount of functional enzyme, respectively (Figure 1C). The adherent mucilage of Dja-1 and Sk-1-1 accessions had a different appearance to that of *mum2* mutants, in agreement with their causal mutations being in *PMEI6* and *PER36*, respectively (Figure S1I and S1J).

### Imbibition is modified in mutants that do not release mucilage

In order to examine in more detail the ecological significance of the absence of mucilage release in Arabidopsis accessions, physiological characteristics were examined in more detail. For these studies, insertion mutants in genes affecting mucilage production were used as the natural variants contain other polymorphisms that could influence the phenotypes examined. Three mucilage-release mutants were used, all in the reference accession Col-0; *mum2-11* accumulates normal amounts of polysaccharides, whereas *myb61* and *myb5-1* have reduced polysaccharide accumulation [6], [25], [33], [34]. No consistent differences in germination were observed for different seed lots of wild-type, *mum2-11* or *myb61* seeds grown on high PEG concentrations (Figure S2; Protocol S2). As seeds for the same genotype from three independent cultures showed different germination capacities at high osmotic potential, this phenotype would appear to vary depending on the environmental conditions of mother plant culture.

The effect of mucilage retention on seed imbibition was examined in more detail for *mum2-11* and *myb5-1*. Water mobility during seed imbibition was analysed by low-field NMR spectroscopy. The proton spin-spin relaxation times (T_2_) depend on their molecular environment. NMR relaxation time measurements carried out on plant tissues are sensitive to the water content and localization of water in cells [35], [36] and relaxation signals are generally described by a multi-exponential behaviour reflecting different water compartments [37], [38]. T_2_ relaxation times were firstly attributed to water associated with different seed compartments using wild-type seeds (Table 2).

**Table 2:** Attribution, value and amplitude of T_2_ relaxation signals obtained in low-field NMR analyses of dry and imbibed Arabidopsis seeds.

	Component														
	1			2			3			4			5		
Signal attribution in dry seeds	Solid phase (macromolecules)			Exchangeable protons			Oil			Oil			-		
Attribution in imbibed (imb) seeds	Solid phase (macromolecules)			Exchangeable protons			Water within seeds and oil			Water within adherent mucilage and oil			Water outside seeds		
	%A(1)	T_2_(1)	water (%)	%A(2)	T_2_(2)	water (%)	%A(3)	T_2_(3)	water (%)	%A(4)	T_2_(4)	water (%)	%A(5)	T_2_(5)	water (%)
**WT Col0 WSM imb 1 h**	**12.5**	**0.0240**	0	**26.2**	**3.487**	19	**29.6**	**43.9**	10.39	**31.8**	**223**	12.52	-	-	-
**WT Col0 dry**	**49.1**	**0.0164**	0	**11.5**	**0.231**	8.46	**20.2**	**35.2**	0	**19.2**	**138**	0	-	-	-
**WT Col0 imb 24 h**	**10.5**	**0.0249**	0	**18.4**	**2.466**	15.59	**33.0**	**19.8**	17.29	**38.1**	**114**	22.16	**9.9**	**457**	8.39
***mum2-11*** ** dry**	**45.6**	**0.0165**	0	**11.2**	**0.211**	8.5	**22.1**	**35.2**	0	**21.0**	**140**	0	-	-	-
***mum2-11*** ** imb 24 h**	**14.0**	**0.0239**	0	**15.4**	**1.068**	12.30	**46.5**	**11.7**	21.05	**20.3**	**78**	0.99	**3.9**	**319**	3.09
***myb5-1*** ** dry**	**42.9**	**0.0165**	0	**10.5**	**0.176**	8.10	**23.9**	**35.8**	0	**22.7**	**141**	0	-	-	-
***myb5-1*** ** imb 24 h**	**11.6**	**0.0231**	0	**15.5**	**1.212**	12.66	**37.3**	**13.5**	15.92	**29.9**	**67**	10.67	**5.7**	**266**	4.61

Water contributions to components 3 and 4 in imbibed seeds were calculated based on oil contents in dry seeds. WSM, without soluble mucilage.

**Figure 2:**
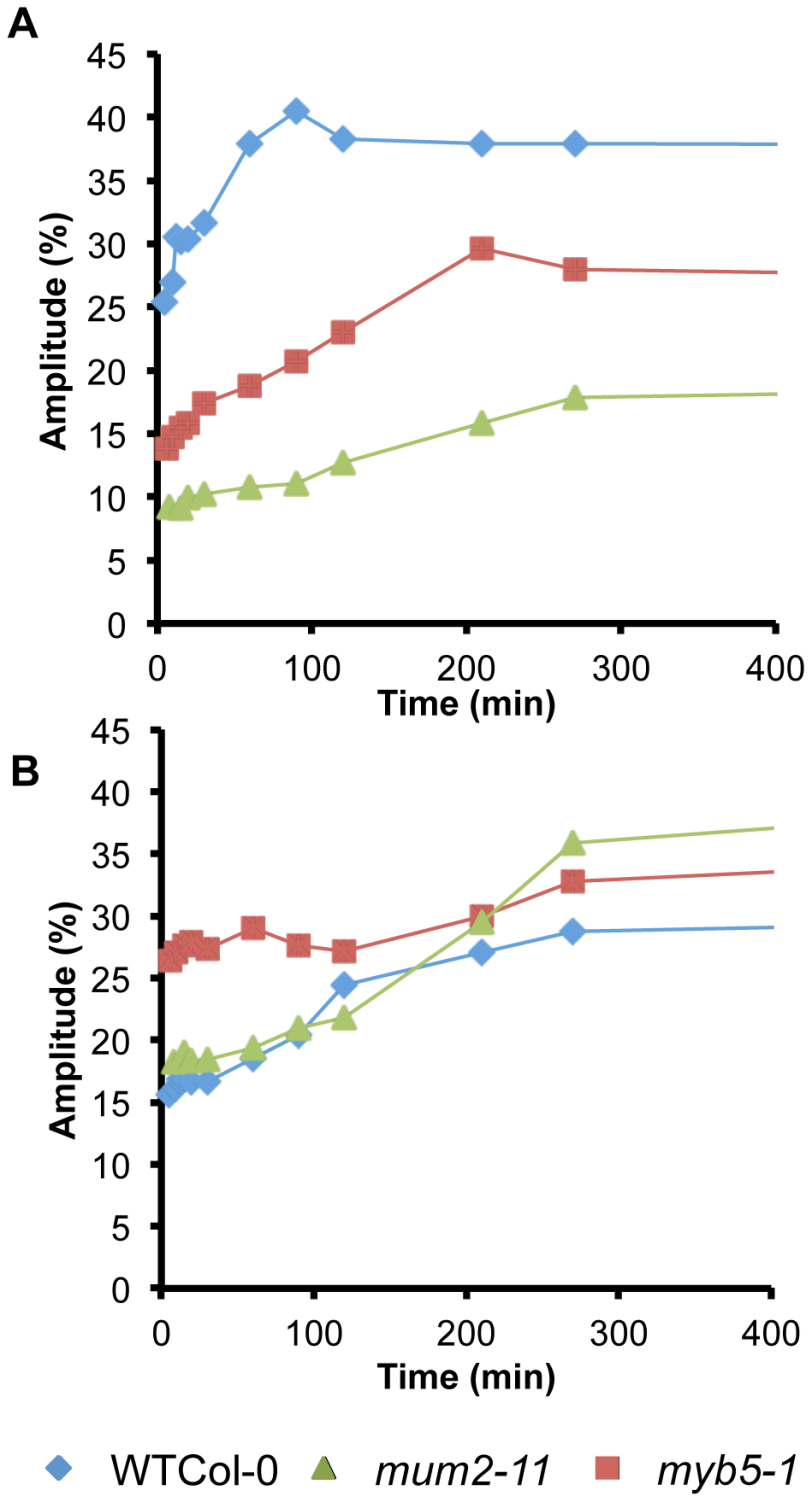
*mum2* seeds float at the water surface. (*A*) After placing in contact with water the majority of *mum2-11* seeds float, in contrast to wild-type Col-0 (WT) seeds that sink to the bottom of the tube. (*B*) Floating *mum2-11* seeds germinate after 48 h of imbibition at the water surface.

Water transfer rates between compartments were determined from the evolution of signal amplitudes. Although, wild-type seeds showed the expected rapid water uptake by mucilage polysaccharides over the first 90 minutes (Figure 2A), water transfer to internal seed tissues was slower for wild-type seeds than in mucilage release mutants (Figure 2B and Figure S3). This indicates that during early imbibition while water is transferred into the *mum2-11* and *myb5-1* seeds to interact with macromolecules, in the wild type it remains trapped outside the seed in mucilage. Furthermore, transfer of water to internal seed tissues was higher in *myb5-1* than *mum2-11*, with amplitude already at high levels after the 5 minutes that had elapsed before the first measurement (Figure 2B). The presence of more non-released mucilage polysaccharides in *mum2-11*, therefore, reduced water transfer inside the seed compared to *myb5-1*.

A previous study has shown that *myb5-1* seeds have higher oil contents than wild type [39]. Determination of seed oil content for dry seeds (Table 2, A(3)+A(4)) confirmed this finding (46.1%±0.71 for *myb5*-1, 40.34%±1.35, wild type and 42.62%±0.73, *mum2-11*).

### Seeds of mucilage release mutants float

Mutations of *MUM2*, *PMEI6* and *PER36* may result in modifications in other tissues where these genes are transcribed. Although *MUM2* is almost exclusively expressed in vascular tissue in vegetative tissues [25], the naturally occurring mutations are unlikely to increase fitness through improved water management as no difference was observed for *mum2* mutants compared to wild type on water deficit (Figure S4; Protocol S3).

**Figure 3:**
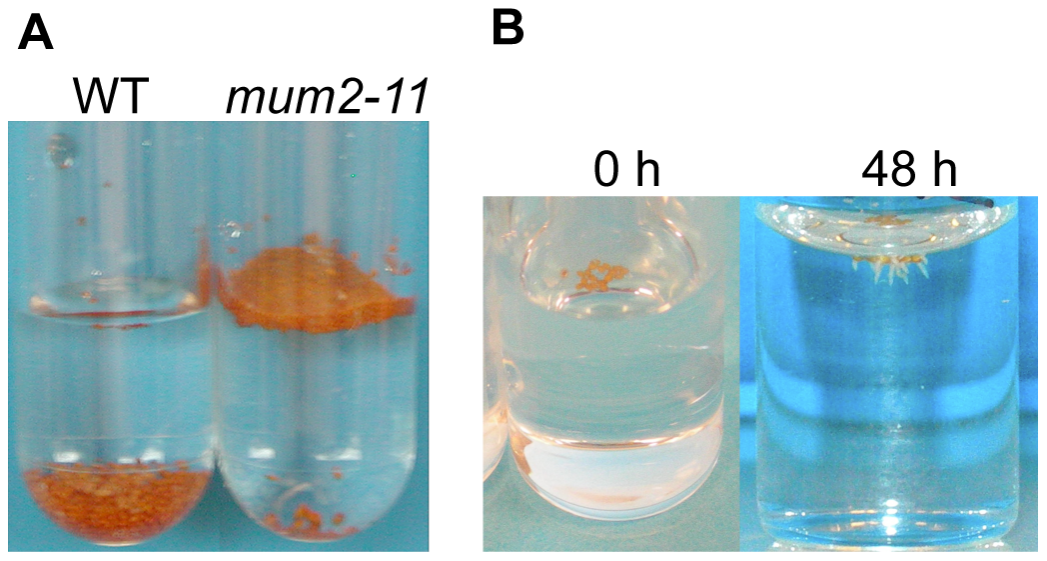
Maintenance of seed buoyancy in *mum2* seeds is associated with delayed water uptake. Evolution of water uptake by adherent mucilage and internal seeds tissues as determined by low-field NMR. Variation of the amplitude of T_2_ values (*A*) for, water in adherent mucilage and oil (component 4), and (*B*) water in internal seed tissues (component 3), during imbibition of seeds of wild type, *mum2-11* and *myb5-1*. Standard errors are estimated at 3.4% and 1%, respectively. Results from two experiments with seeds from independent cultures gave similar results. WT, wild type.

Dry seeds of *Plantago coronopus*, a myxospermous species that grows in desert highlands, can be dispersed by run-on rainwater before sinking and adhering to soil by released mucilage [40]. In a similar manner, it was noted that when water was added to seeds of the *mum2-11* mutant or accessions that do not release mucilage, the majority of seeds floated at the surface, whereas most wild-type Col-0 or mucilage releasing seeds sank (Figure 3A). Wild-type Col-0 seeds floated for a few seconds before mucilage was released and increased seed specific weight, whereas mutant seeds continued to float for many hours and even germinated at the water surface (Figure 3B) confirming that floating seeds were imbibed. The floating phenotype was also observed for seeds of *myb5*, *ttg1*, *gl2* and *mum4* mucilage release mutants where mucilage accumulation is reduced [22], [24], [33], [34], [41]. Buoyancy maintenance was not due to differences in mutant dry seed surface area or weight (Figure S5; Protocol S4).

### Identification of a new class of natural mucilage variants that float

**Figure 4:**
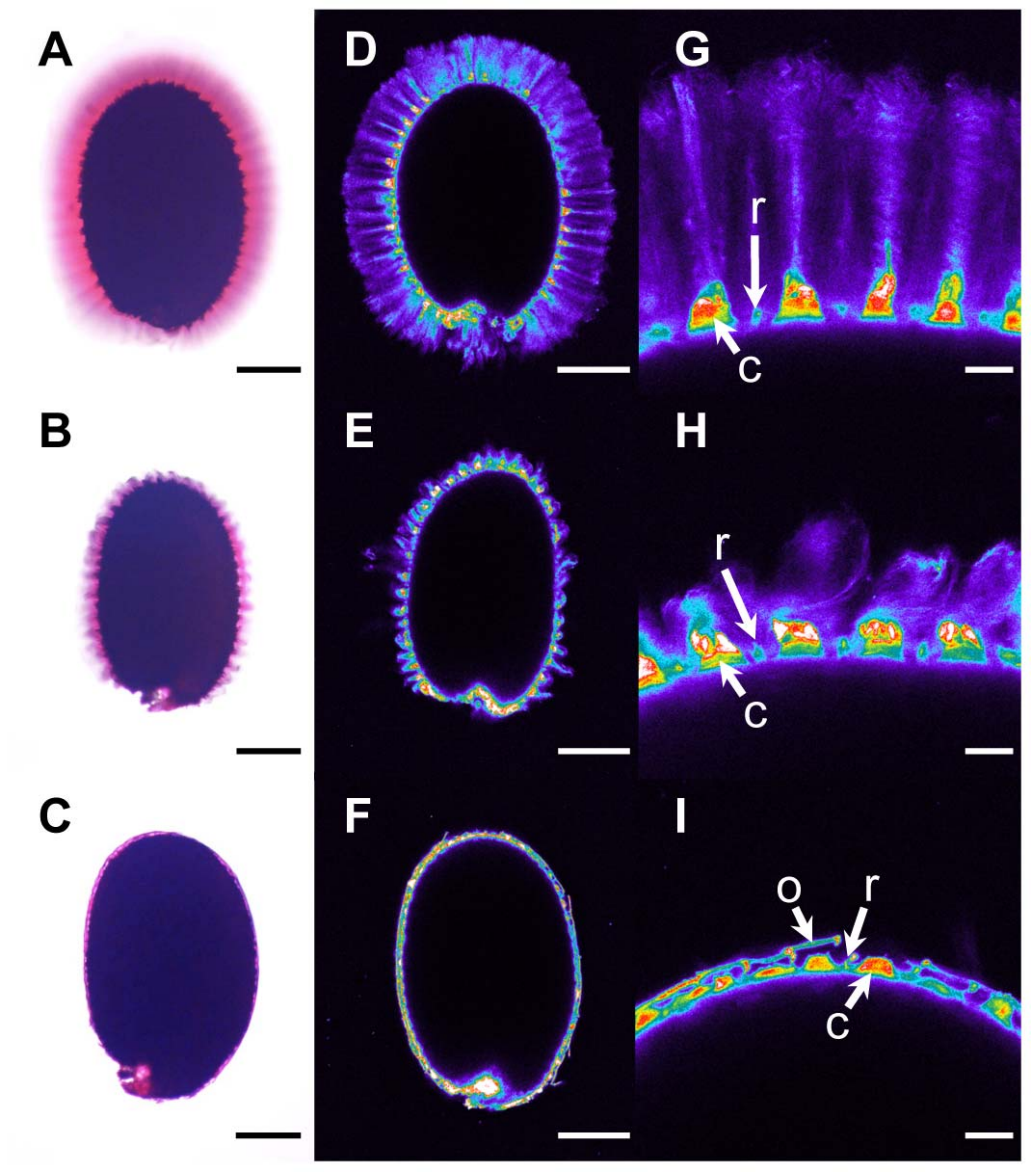
Adherent mucilage width and cellulose labelling are reduced in floating mucilage-releasing (FMR) accessions. Ruthenium red staining of pectins (*A*) to (*C*) and Pontamine S4B staining of cellulose (*D*) to (*I*) in the adherent mucilage released from imbibed seeds of wild-type Col-0 (*A*), (*D*) and (*G*), *cesa5-1* (*B*), (*E*) and (*H*), and the FMR accession Rak-1 (*C*), (*F*) and (*I*). (*G*) to (*I*) are magnifications of regions in (*D*) to (*F*), respectively. Images (*D*) to (*G*) are shown using the Rainbow2 look-up table. Bars = 150 µm (*A*) to (*F*) or 20 µm (*G*) to (*I*). C, Columella; o, outer cell wall; r, radial cell wall.

Since flotation was observed in central Asian and Scandinavian accessions, we screened for seed flotation in an extra set of 53 Arabidopsis accessions from central Asia (including Russia) and Scandinavia (Table S4). Seeds were examined for flotation by imbibition in water followed by ruthenium red staining; seeds of four accessions floated and these originated from the Altaï Republic of Russia, close to the border with Kazakhstan. Interestingly, staining indicated that mucilage had been released from these accessions, and that the adherent layer of mucilage was greatly reduced (Figure 4C compared to 4A); these accessions were termed Floating Mucilage Releasing (FMR) accessions.

Cellulose in adherent mucilage is necessary for fixing mucilage pectin domains to the seed; seeds of *cesa5*, *fei2* and *sos5* mutants are affected in cellulose production and have a reduced amount of adherent mucilage (Figure 4B) [10]–[12]. Imbibed seeds of FMR accessions showed little or no cellulose labelling within the thin layer of adherent mucilage, in contrast to the wild-type Col-0 accession where cellulose was visible as rays originating from the tops of columella as well as diffuse labelling between rays (Figure 4D and 4G). Labelling of FMR seeds was even more reduced than that observed with *cesa5* (Figure 4E and 4H). Nevertheless, in the FMR accessions columella and radial cell walls were visible, as well as outer cell wall fragments (Figure 4F and 4I).

**Figure 5:**
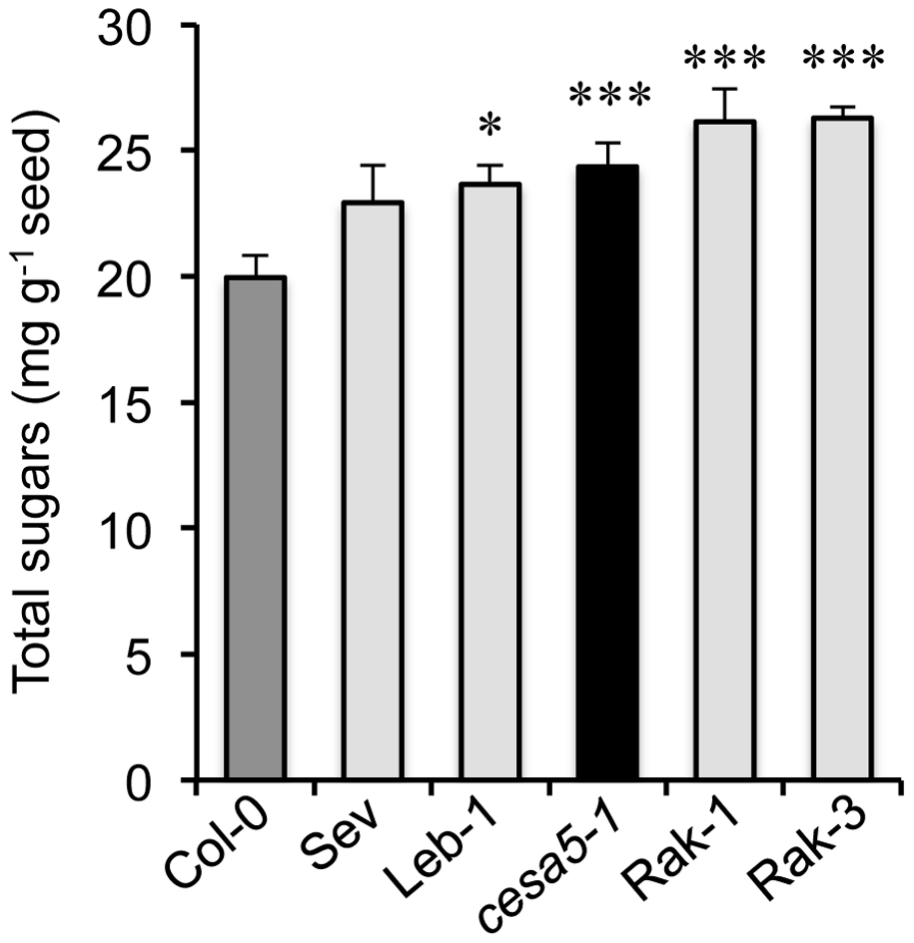
Floating mucilage-releasing (FMR) accessions have higher water-soluble mucilage sugar contents than wild type. Amounts of sugars in water-soluble mucilage for wild-type Col-0 (Col-0; dark-grey bar), *cesa5-1* (black bar), and FMR accessions (light-grey bars). Values are means of four independently extracted samples from two biological repeats. Error bars represent SE (n = 4). Mann-Whitney *U* test with wild-type Col-0, *P<5%, ***P<0.1%.

The reduction of adherent mucilage in the *cesa5* mutant has been associated with a redistribution of RG I sugars to the water-soluble mucilage layer [12]. The amount of sugars in water-soluble mucilage was, therefore, determined for the different FMR accessions compared to wild-type Col-0 and *cesa5-1* (Figure 5). All four FMR accessions had higher amounts of water-soluble mucilage sugars than wild type, like *cesa5-1*, indicating that FMR mutants also show redistribution of mucilage to the water-soluble layer.

## Discussion

Natural variation between populations is a useful tool for the identification of mutations that produce physiological changes. Previously a visual screen for defective mucilage release from seeds of Arabidopsis accessions had identified Shahdara and Dja-1 as natural mutants affected in the *MUM2* and *PMEI6* genes, respectively [25], [29]. Here we have identified a further eight accessions affected in mucilage release and shown that their seeds float on water (Table 1; Table S1; Figure 3). An additional four accessions were also identified whose seeds float, but release mucilage, termed FMR accessions (Figure 4; Table S4).

Seven of the eight accessions affected in mucilage release were *mum2* mutants resulting from six different mutations that were distinct from the original Shahdara mutation (Figure 1A). In the accessions that are *mum2* mutant alleles, *MUM2* transcript abundance and the extent of adherent mucilage swelling were in accord with the mutations identified (Figure 1B and 1C; Figure S1). The Sk-1-1 accession was found to contain a non-sense mutation in *PER36*. The identification of only one natural mutant in *PMEI6* and *PER36* genes compared to eight variants with 7 causal mutations for *MUM2* may be due to the size of the *MUM2* gene region, 5751 bp, which would increase the probability of mutation compared to the 816 bp and 1451 bp of *PMEI6* and *PER36*, respectively.

FMR accessions had an extremely reduced layer of adherent mucilage (Figure 4C), whereas soluble mucilage amounts were higher than those of the Col-0 accession (Figure 5). Mutants affected in the synthesis of cellulose present in adherent mucilage, *cesa5*, *fei2* and *sos5*, have similar mucilage phenotypes. Nevertheless, some cellulose is still observed in the adherent mucilage of these mutants as rays, even in knockout mutants (Figure 4E and 4H) [10]–[12], and these are not observed in the FMR accessions (Figure 4F and 4I). Furthermore, seeds of *cesa5-1* mutants do not float. This indicates that the mutation(s) in FMR accessions have a more profound effect on the synthesis of cellulose present in seed mucilage. Identification of the gene(s) affected should further our understanding of cellulose production and the interactions between cellulose and pectin.

Seed flotation enables long-distance dispersion on water. A previous study has observed seed dispersion by flotation on run-on rainwater for *Plantago coronopus*, which inhabits desert highlands. This species also releases mucilage from seeds and it was observed that dry seeds floated for between 10 to 44 minutes before sinking [40]. Similarly mucilage retention in the seed coat, or its release as mainly water-soluble mucilage, allowed Arabidopsis seeds to float, and even germinate on the water surface (Figure 3). Mutations causing natural Arabidopsis variants to float could, therefore, be a local adaptation to improve seed dispersal. In effect, the collection sites of Neo-3, Neo-6 and Sus-1 accessions were near to rivers. The external surfaces of seed coats are remarkably diverse and features such as hairs or wings are often used to assist dispersal. Genetic variation in Arabidopsis seed dispersal has previously been observed, but was a maternally inherited trait controlled by plant architecture and expressed in high-density populations [42], [43]. This contrasts with the genetic variants identified here that might modify seed dispersal independently of maternal effects. Although the existence of genetic variation for a characteristic that could influence dispersal reveals the potential for evolution of this trait, the advantage of floating due to modified mucilage properties remains to be demonstrated; at the species level, the footprint of natural selection was not observed.

Seed mucilage polysaccharides represent 3% of dry seed weight [12] and their production is a significant metabolic investment for the mother plant. Several mucilage release mutants have been identified that accumulate reduced amounts of mucilage and tests using *myb5*, *ttg1*, *gl2* and *mum4* mutants showed that their seeds also floated. Yet, none of the accessions affected in mucilage release were affected in loci that reduce the accumulation of mucilage polysaccharides (Figure 1C) [25], [29]. This suggested that the non-released polysaccharides present in the epidermal cells of the seed coat serve a function. Low-field NMR analysis of water uptake by seeds showed that rates of transfer inside the seed and in interaction with macromolecules in seed tissues were higher in *myb5-1* than *mum2-11* (Figure 2; Figure S3). The presence of more mucilage polysaccharides in the epidermal cell layer, therefore, decreased the speed with which the tissues within the seed were imbibed and would maintain *mum2-11* seed buoyancy, whereas the floating capacity of *myb5* seeds would be reduced.

The four FMR accessions present a novel mucilage phenotype with almost no adherent mucilage (Figure 4) while the amounts of soluble mucilage released were higher than those observed in wild-type Col-0 (Figure 5). In these accessions, the mucilage produced could not maintain buoyancy by reducing water uptake by internal seed tissues. Mucilage is also produced by root cap cells and, like seed coat mucilage, it is at the interface between plant cells and the external environment. One of the roles proposed for root cap mucilage has been the structuration of bacterial communities present in the rhizosphere through its metabolism [44]. In the same manner, a recent study has implicated the metabolism of seed mucilage by soil microorganisms in the promotion of *Artemisia sphaerocephala* seedling growth [45]. The soluble mucilage from hydrated seeds of FMR accessions would be released into the soil near the mother plant and could aid in the modulation of soil flora as observed for root mucilage [reviewed in 45].

Released mucilage has been proposed to enhance seed hydration and to enable germination under conditions of reduced water potential [6]. Although rapid uptake of water was observed for adherent mucilage polysaccharides in wild-type Col-0 seeds (Figure 2A), water was transferred at a slower rate to internal seed tissues compared to seeds of mucilage release mutants (Figure 3B; Figure S3). This demonstrates that although mucilage polysaccharides are an excellent hydrogel, they do not increase transfer of water into seeds. Accordingly, seed germination on high concentrations of PEG was not consistently different between seeds that released mucilage and those that did not (Figure S2). The more rapid imbibition of internal seed tissues in mucilage release mutants might enable them to germinate more rapidly, but differences in germination reported for certain Arabidopsis mucilage mutants have indicated that the absence of released mucilage causes a germination lag [6], [18]–[20]. Alternatively, as the adherent mucilage traps water around the seed, this could slow the rate of seed drying. Studies on the desert shrub *Artemisia sphaerocephala* have shown that DNA repair was improved in achenes with mucilage humidified by desert dew, compared to achenes where mucilage had been removed [46]. Seed mucilage could, therefore, prolong the imbibed state, providing more time for repair mechanisms to function and thereby improve seed longevity.

In conclusion, the identification of fourteen natural Arabidopsis mucilage mutants that float has highlighted the occurrence of at least ten independent mutation events, affecting four different loci, in populations in central Asia and Scandinavia. These mutations lead to modifications in seed mucilage production that maintain buoyancy compared to seeds that release a thick adherent layer of mucilage. Whether this is an adaptive trait requires further study, as among the accessions studied here the population genetic signature of local adaptation was not detected. Genetic variation in mucilage properties promises to contribute to our understanding of the ecological relevance of its production and the populations where mucilage release is not a fixed trait will be an important tool for testing potential roles.

## Materials and Methods

### Plant material and mucilage phenotyping

Accessions were obtained from the Versailles Arabidopsis Stock Centre (http://publiclines.versailles.inra.fr/) except for the Norwegian and Swedish populations in Table 1 and Table S1, which were kindly donated by Odd-Arne Rognli through NARC (Norway). Seeds for new central Asia accessions, including Dja, Sus and Neo, were collected from their natural habitat (Table S1) http://www.inra.fr/vast/collections.htm). Genetic differentiation of *MUM2* was examined in 289 lines corresponding to 15 Norwegian, 6 central Asian, 7 Spanish and 13 French populations [32]. The *mum2-10*, *mum2-11* and *cesa5-1* mutants (SALK_011436, SALK_110461 and SALK_125535, respectively; Col-0 background) were obtained in previous studies [25], [47]. The *ttg1* (GK_286A06), *mum4* (SALK_085051), *myb61* (SALK_106556) and *gl2* (SM_3_16350) mutants, all Col-0 background, were obtained from the Nottingham Arabidopsis Stock Centre ([48]–[50]; http://arabidopsis.info). The *myb5-1* (SALK_030942) and *aba3-1* mutants [51] were a gift of C. Dubos and M. Koornneef, respectively. Crosses were performed between *mum2-12* (Shahdara accession) and Dja-1, Sus-1, Neo-3, Neo-6, Ale-8, Had-3-1, Lom-3-1, Nfro-1-1, Sk-1-1 and Sku-20, and between Dja-1 and Sk-1-1 accessions. As the seed coat is maternally derived, seed coats of progeny from F_2_ and F_3_ generations were examined; only seeds from crosses with Dja-1 and Sk-1-1 showed genetic complementation. The gene affecting mucilage release in Sk-1-1 was localised to an interval on the lower arm of chromosome 3 using a mapping population of 176 F2 individuals derived from a cross between Col-0 and Sk-1-1 accessions. DNA was extracted from 33 F2 progeny that did not release mucilage, using 10 seedlings from each. Whole-genome resequencing was carried out on this DNA by Genome Enterprise Ltd (http://www.norwichresearchpark.com/parkdirectory/genomeenterpriselimited.aspx) using an Illumina HiSeq 2000 sequencer. The SHOREmap software package with bwa aligner (http://1001genomes. org/downloads/shore.html) was used to map the Sk-1-1 polymorphisms against those of the Col-0 accession and identify mutations. The output of this pipeline was filtered for mutations in the mapping interval on the chromosome 3. These polymorphisms were then filtered against polymorphisms present in the genomes of 17 accessions available from the 1001 genome project (http://1001genomes.org/index.html) and known to release mucilage; Alc-0, Altai-5; Baz-0, Blh-1, C24, Dja-1, Gol-1, Jea, Ler-0, Neo-6, Oy-0, Qar-8a, Ri-0, Sakata, Sus-1, Ws-2 and Zal-1. These sequence data were produced by the Weigel laboratory at the Max Planck Institute for Developmental Biology, the Ecker laboratory at the Salk Institute [52], the Pennacchio laboratory at the DOE Joint Genome Institute, and the Mott laboratory at the Wellcome Trust Center for Human Genetics [53]. Of the 258 SNPs that were Sk-1-1 specific only 4 resulted in the introduction of a stop codon; one of these was in *PER36*, which has been shown to be required for mucilage release [30].

All phenotypic analyses were carried out using seed lots or tissue obtained from plants cultured simultaneously. Phenotyping for mucilage release was carried out as described previously [25] or by imbibing seeds on filter paper (6 cm dia.) hydrated with 700 µL of water for 1 h followed by staining with 200 µg mL^−1^ ruthenium red. Wild-type Col-0, *mum2-11* and accessions defective for release were treated with 0.05 N HCl then 0.3 M NaOH to release mucilage and stained with ruthenium red as described previously [25].

Seeds for 53 accessions (Table S4) were obtained from 2 independent series of plants cultured in compost (Tref Substrates, http://www.jiffygroup.com/) in a growth chamber (21°C day, 17°C night, 150 µmol m^−2^ s^−1^ light intensity, 16 h photoperiod, 65% relative humidity). A seed lot from each culture was examined for seed flotation by imbibition of seeds for 10 min in water, followed by staining in 500 µg mL^−1^ ruthenium red and observation with a light microscope (Axioplan 2; Zeiss; http://www.zeiss.fr/).

### DNA extraction and obtaining *MUM2* sequences

DNA for PCR analysis was extracted from flower buds as described by Doyle and Doyle [54]. The full sequence of *MUM2* in a subsample of 28 accessions was retrieved from public databases [55]. Otherwise, sequencing of the *MUM2* gene was performed as described by Sullivan et al. [12]. The *Arabidopsis lyrata* sequence of *MUM2* was obtained from the public database and used as outgroup (http://www.phytozome.net/alyrata) [56]. Genotyping with the *MUM2* ^Sha^ marker was carried out by PCR amplification of either a 162 bp (wild-type) or 118 bp (*mum2-12*) DNA fragment with forward primer 5′-TGGTCGTTATTGGGTCTCGT-3′ and reverse primer 5′-TTAAGAACGCCCGAGGAATA-3′. Two fragments of *MUM2* were re-sequenced for 41 Arabidopsis accessions collected worldwide and for the 110 individuals collected in Norway and central Asia; the 5′ portion of ∼1000 bp was amplified using forward primer 5′-GAAGGAGGCATCGATGTGAT-3′ and reverse primer 5′-GGTGAGTTTGGTCCAGGAAA-3′ and the 3′ portion of ∼600 bp was amplified using forward primer 5′-CTGGAGCTTACATGGAGAGGA-3′ and reverse primer 5′-CAAGAGGATCACCTTCC-3′.

### Expression analysis

Developing siliques at 8 to 12 days after pollination were pooled and total RNA extracted and reverse transcribed as described previously [29]. Quantitative real-time PCR reactions were performed as described by Plessis et al. [57]. *MUM2* specific primers were as follows, forward primer 5′-CAGCGGCATGGTTGGTCT-3′ and reverse primer 5′-CCAAGCAAACCCACCGAGT-3′, and had been tested for their efficiency rates and sensitivity on a dilution series of cDNAs. *EF1α4* primers have been described previously [57].

### Extraction and analysis of water-soluble mucilage

Water-soluble mucilage extracts were obtained from 200 mg of intact seeds as described previously [12] The uronic acid (as GalA) and total neutral sugar (as Rha) contents were determined by the automated m-hydroxybiphenyl and orcinol methods, respectively [58], [59].

### Low-field NMR

^1^H NMR measurements were performed using a Time-Domain spectrometer (Minispec BRUKER; http://www.brukeroptics.com/) operating at a resonance frequency of 20 MHz. The NMR system was equipped with a temperature control device connected to a calibrated optic fibre (Neoptix Inc.; http://www.neoptix.com/) allowing for ±0.1°C temperature regulation. For the assignment of T_2_ relaxation times to different water compartments, measurements were firstly obtained for three different wild-type Col-0 samples; intact imbibed seeds, intact dry seeds and imbibed seeds pre-treated to remove soluble mucilage, but with adherent mucilage; water-soluble mucilage was extracted from wild-type Col-0 seeds as described previously [12] and after lyophilisation, seeds without soluble mucilage were resuspended in water and analysed by NMR. The mature, “dry” Arabidopsis seeds (approximately 8% water content) were introduced into NMR tubes (10 mm dia.). In order to fill tubes to a 10 mm height, corresponding to the homogeneous region of the NMR radiofrequency, 200 to 220 mg of Arabidopsis seeds were used and imbibed with 150 to 220 µl of deionised water, tubes were then weighed and hermetically sealed. Acquisitions of T_2_ were carried out from 5 min to 24 h of imbibition and samples were analysed at ambient temperature (20°C). Two types of pulse sequences were used; proton free induction decays (FID) were acquired using the following parameters: a 90° pulse of 2.6 µs, a dwell time of 0.4 µs between two successive data points, 16 scans of 150 data points, and a recycle delay of 9 s between each scan, and the Carr–Purcell–Meiboom–Gill (CPMG) pulse sequence with a time echo of 0.2 ms. Sixteen scans were acquired for each genotype with 8000 or 16000 data points [60].

Transverse relaxation data were analysed with the following model:
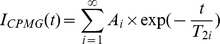
where T_2i_ are the relaxation times of the mobile populations and A_i_ is the intensity of the mobile populations [60]. To ensure the accuracy of the data treatment, spin–spin relaxation decay curves were fitted using MEM [61] and a discrete method [62].

In imbibed intact seeds five T_2_ components were identified which could be assigned to protons of water populations with different mobility and ratio (expressed in relative percentage) (Table 2). The first and second components, T_2_(1) and T_2_(2), were also observed in dry seeds and could be assigned, respectively, to the solid phase of the seed, notably macromolecules such as polysaccharides and proteins, and protons in exchange with the hydroxyl groups found in these macromolecules. The latter two components (T_2_(3) and T_2_(4)) were assigned to oil with a superposed contribution from water in both as intracellular water in the former and in adherent mucilage in the latter. Comparison of T_2_(3) and T_2_(4) values for dry seeds with those obtained in imbibed seeds with water-soluble and adherent mucilage or only adherent mucilage confirmed the latter attributions (Table 2). A fifth component, T_2_(5), was identified in imbibed wild-type seeds that corresponded to water in soluble mucilage. This component was also present in imbibed mutant seeds where it was attributed to water outside seeds (Table 2).

In order to verify the assignment given to the multi-exponential NMR signals for water in seed compartments, at the end of NMR measurements, the water content of samples was estimated by weighing before and after drying in an oven at 103°C for 24 h. The amplitudes of the NMR signals from the seeds before and after imbibition were compared to the amplitude of the signal expected from distilled water. The signals expected from water were calculated for each sample and each compartment as the product of its mass, its water content and the signal of the distilled water per unit mass. The water content of each compartment was then calculated by deducing the signal contribution from oil.

Analysis of the evolution of signal amplitudes for the different components allowed the water transfer rates between different compartments to be determined. The total water content was calculated using the T_2_ amplitude and was coherent with results using a gravimetrical method before and after drying seeds. Calculation of water content for each compartment confirmed that even after 24 h of imbibition, approximately 40% of water associated with wild-type seeds was trapped outside in mucilage (Table 2).

### Cytochemical staining of adherent mucilage

Seeds were imbibed in water for 3 h then rinsed twice prior to staining. The released adherent mucilage was stained with ruthenium red as previously described [25] or Pontamine Fast Scarlet S4B in 150 mM NaCl [63]. Observations were carried out with a light microscope for ruthenium red (Axioplan 2; Zeiss) or a Zeiss LSM710 confocal microscope using a 561 nm diode laser line to excite Pontamine and detecting fluorescence emission between 570 and 650 nm. For comparison of signal intensity within a given experiment laser gain values were fixed.

## Supporting Information

Figure S1 Adherent seed mucilage of natural *MUM2* mutants. Ruthenium red stained seeds of (*A*) wild-type Col-0, (*B*) *mum2-11*, (*C*) Shahdara, (*D*) Ale-8, (*E*) Neo-6, (*F*) Sku-20, (*G*) Sus-1, (*H*) Had-3-1, (*I*) Dja-1 and (*J*) Sk-1-1 after mucilage release by sequential treatment with HCl and NaOH. Images outlined in blue correspond to central Asian accessions and in pink to Scandinavian accessions. Scale bars, 150 µm.
(PDF)

Figure S2 Germination at high osmotic potential is not modified in the *mum2-11* mutant. Germination of *mum2-11* seeds on PEG 8000 for seeds from three independent cultures, (*A*), (*B*) and (*C*), do not show consistent differences compared to wild-type Col-0 and *myb61*. WT, wild type.
(PDF)

Figure S3 Amplitude evolution of T_2_ for water in exchange with macromolecules (solid lines) and for macromolecule protons in seed tissues (dotted lines) during imbibition as determined by low-field NMR. Standard errors are estimated at 0.5%, respectively. Two independent experiments gave similar results. WT, wild type.
(PDF)

Figure S4 Water loss is not modified in *mum2* mutants. (*A*) Rapid dehydration of *mum2-11* rosettes compared to wild type and the ABA-deficient mutant *aba3-1*. Water loss is expressed as a percentage of the initial fresh weight (FW). Error bars represent SE values (n = 4). (*B*) False colour infrared image of the temperature of drought stressed plants. Plants were 16-days old and watering had been withheld for 3 days. Scale indicates leaf temperature (°C). (*C*) Water content (WC) of three-week old plants that had been watered compared to those where water had been withheld for 7 days. Water contents were calculated as the % (w/w) of rosette weight corresponding to water, as determined from plant weight before and after freeze-drying. Error bars represent SE values (n = 4). WT, wild type.
(PDF)

Figure S5 Dry seed weight and size are unaffected in *mum2* and *myb5* mucilage mutants. (*A*) Seed weight was determined using batches of 50 seeds. (*B*) Seed size was measured as visible surface area. Error bars represent SE (A, n = 12; B, n = 120). Results from three experiments with seeds from independent cultures gave similar results. WT, wild type.
(PDF)

Protocol S1 Analysis of genetic differentiation in *MUM2*.
(PDF)

Protocol S2 Germination assay.
(PDF)

Protocol S3 Water loss assays and Infrared thermography.
(PDF)

Protocol S4 Measurement of dry seed size and weight.
(PDF)

Table S1 Individuals characterized in detail from central Asian and Scandinavian populations affected in mucilage release.
(PDF)

Table S2 Polymorphisms present in the *MUM2* gene between accessions affected in mucilage release.
(PDF)

Table S3 Analysis of geographical variation in Arabidopsis mucilage.
(PDF)

Table S4 Central Asian and Scandinavian accessions analysed for seed flotation.
(PDF)
