# Supplementary figures and images for: Local Evolution of Seed Flotation in Arabidopsis
Source: PLoS Genet. 2014 Mar 13;10(3):e1004221. doi: 10.1371/journal.pgen.1004221 (PMC3953066; doi:10.1371/journal.pgen.1004221)

Figure S1

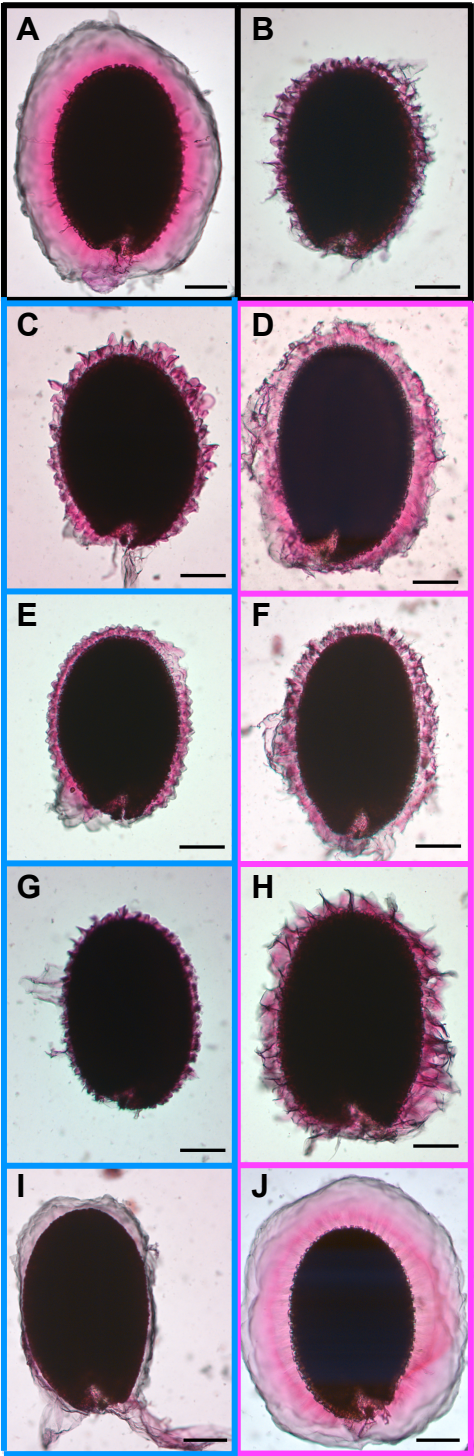

Supplement: Figure S1 — Adherent seed mucilage of natural MUM2 mutants. Ruthenium red stained seeds of (A) wild-type Col-0, (B) mum2-11, (C) Shahdara, (D) Ale-8, (E) Neo-6, (F) Sku-20, (G) Sus-1, (H) Had-3-1, (I) Dja-1 and (J) Sk-1-1 after mucilage release by sequential treatment with HCl and NaOH. Images outlined in blue correspond to central Asian accessions and in pink to Scandinavian accessions. Scale bars, 150 µm. (PDF) [file pgen.1004221.s001.pdf]

Figure S2

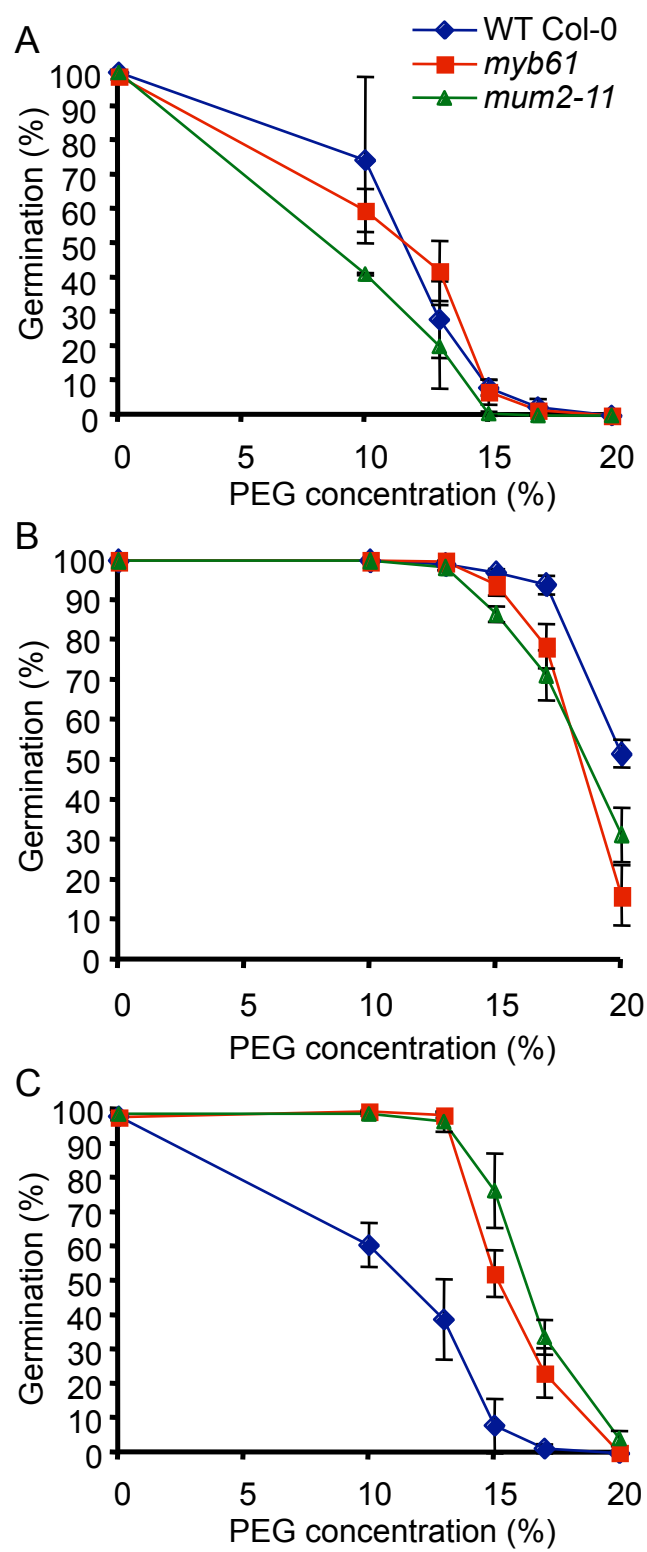

Supplement: Figure S2 — Germination at high osmotic potential is not modified in the mum2-11 mutant. Germination of mum2-11 seeds on PEG 8000 for seeds from three independent cultures, (A), (B) and (C), do not show consistent differences compared to wild-type Col-0 and myb61. WT, wild type. (PDF) [file pgen.1004221.s002.pdf]

Figure S3

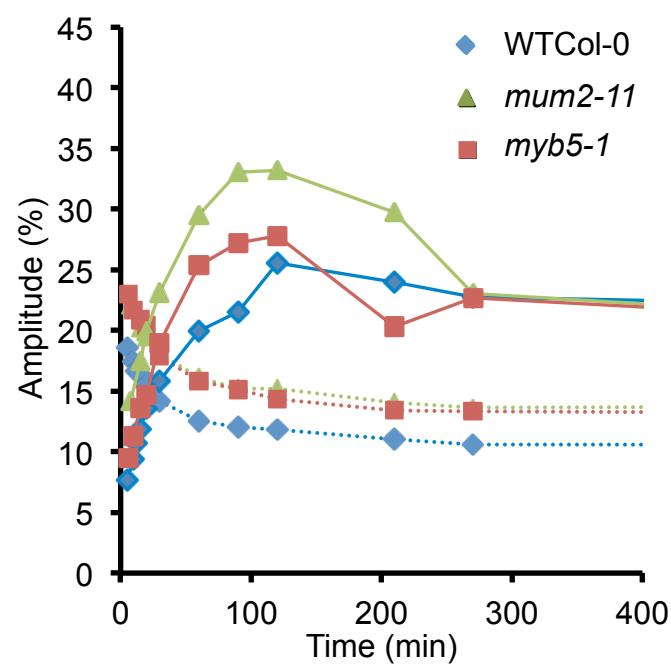

Supplement: Figure S3 — Amplitude evolution of T2 for water in exchange with macromolecules (solid lines) and for macromolecule protons in seed tissues (dotted lines) during imbibition as determined by low-field NMR. Standard errors are estimated at 0.5%, respectively. Two independent experiments gave similar results. WT, wild type. (PDF) [file pgen.1004221.s003.pdf]

**Figure S4**

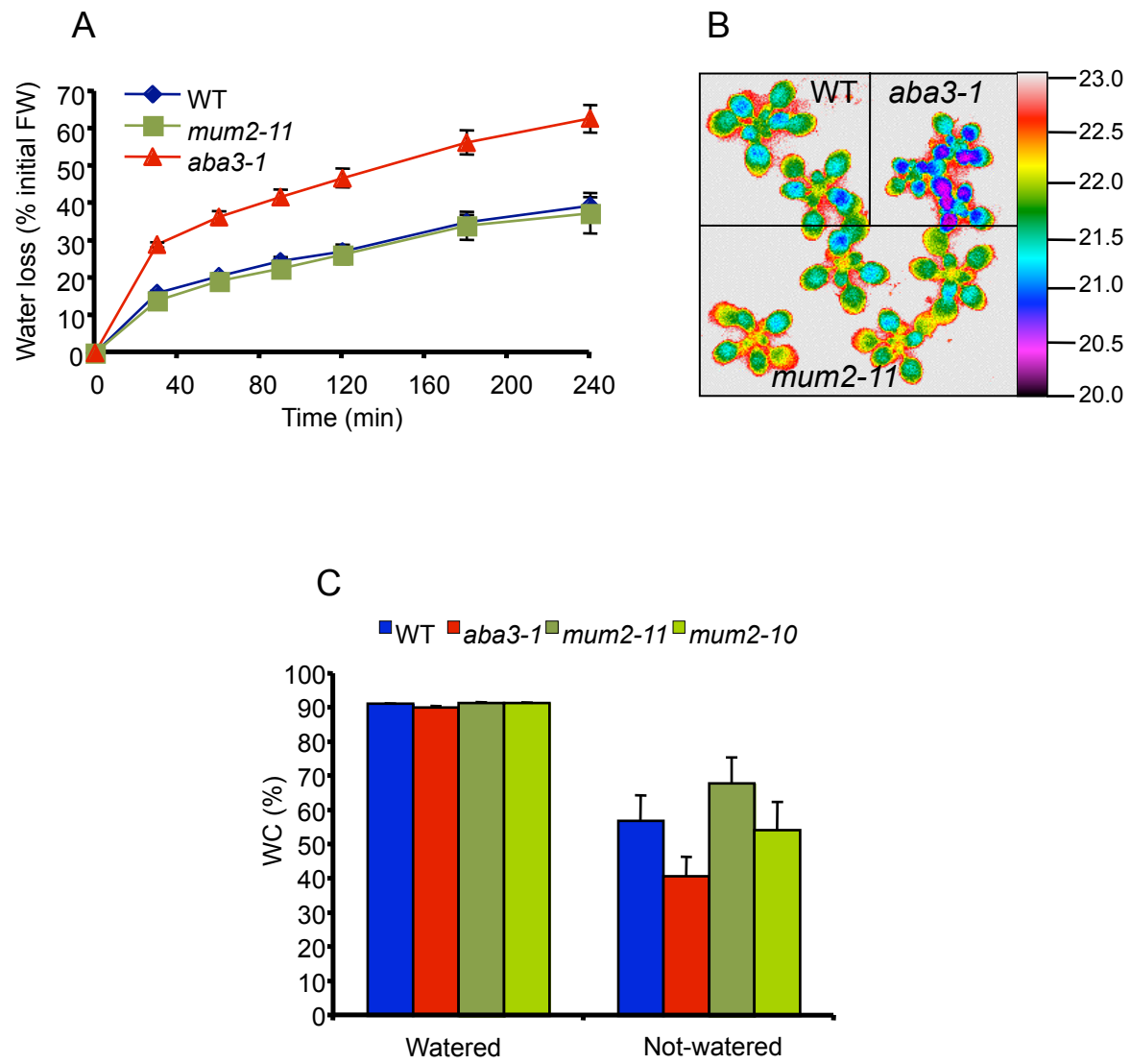

Supplement: Figure S4 — Water loss is not modified in mum2 mutants. (A) Rapid dehydration of mum2-11 rosettes compared to wild type and the ABA-deficient mutant aba3-1. Water loss is expressed as a percentage of the initial fresh weight (FW). Error bars represent SE values (n = 4). (B) False colour infrared image of the temperature of drought stressed plants. Plants were 16-days old and watering had been withheld for 3 days. Scale indicates leaf temperature (°C). (C) Water content (WC) of three-week old plants that had been watered compared to those where water had been withheld for 7 days. Water contents were calculated as the % (w/w) of rosette weight corresponding to water, as determined from plant weight before and after freeze-drying. Error bars represent SE values (n = 4). WT, wild type. (PDF) [file pgen.1004221.s004.pdf]

Figure S5

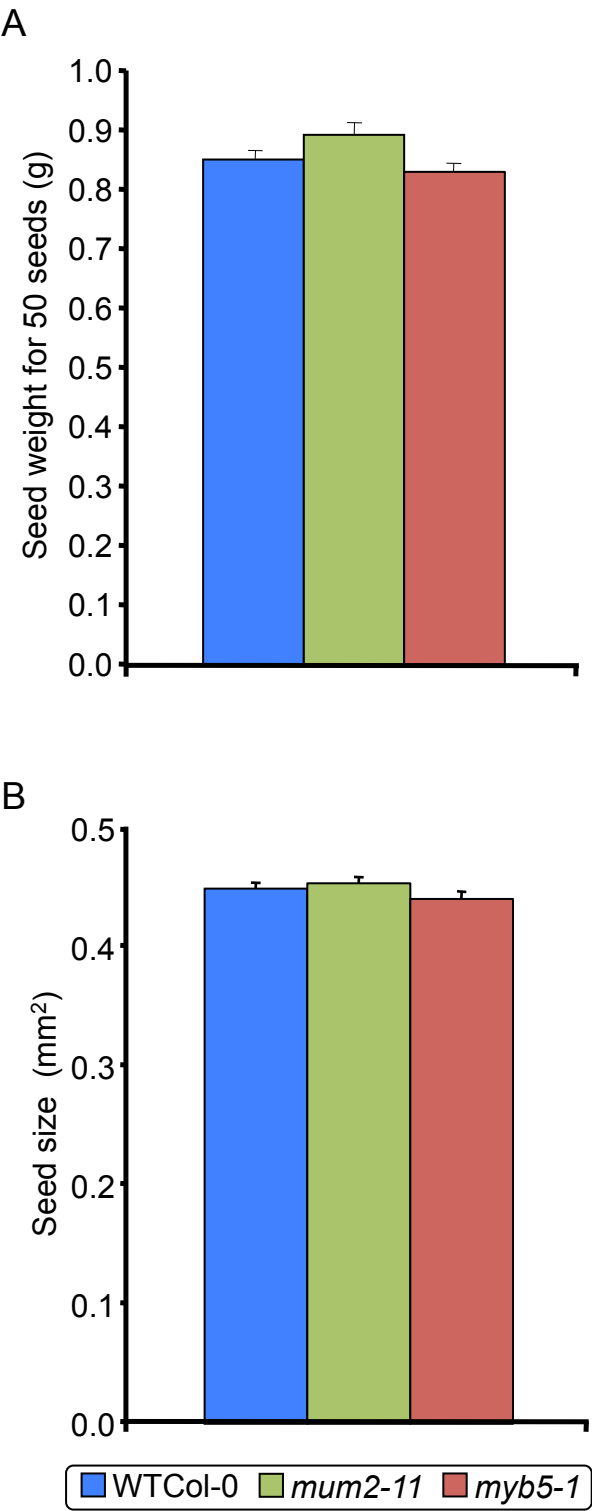

Supplement: Figure S5 — Dry seed weight and size are unaffected in mum2 and myb5 mucilage mutants. (A) Seed weight was determined using batches of 50 seeds. (B) Seed size was measured as visible surface area. Error bars represent SE (A, n = 12; B, n = 120). Results from three experiments with seeds from independent cultures gave similar results. WT, wild type. (PDF) [file pgen.1004221.s005.pdf]
