# Supplementary material for: Local Evolution of Seed Flotation in Arabidopsis
Source: PLoS Genet. 2014 Mar 13;10(3):e1004221. doi: 10.1371/journal.pgen.1004221 (PMC3953066; doi:10.1371/journal.pgen.1004221)
Supplement: Protocol S1 — Analysis of genetic differentiation in MUM2. (PDF) [file pgen.1004221.s006.pdf]

**Protocol S1** Analysis of genetic differentiation in *MUM2*

A total of 289 lines collected from 15 Norwegian, 6 Central Asian, 7 Spanish and 13 French populations, all previously described in [32], were phenotyped for the presence or absence of mucilage. These four broad regions are genetically differentiated [32] and reflect the species history of range expansion. The absence or presence of mucilage was considered as a binary trait. For the analysis of *MUM2* differentiation, we focused on the 6 Central Asian and 15 Norwegian populations. We used primers muc1-F (5'-GAAGGAGGCATCGATGTGAT) and muc1-R (5'-GGTGAGTTTGGTCCAGGAAA) to amplify approximately a 1000 bp fragment in the 5' part of *MUM2*, including exons 3, 4 and 5 and introns 2, 3, 4 and 5. Genetic differentiation was quantified for the mucilage phenotype and the *MUM2* gene and compared to the genetic differentiation observed at 20 microsatellite and 137 single nucleotide polymorphism (SNP) markers described previously [32]. Following recommendation from Kronholm et al. [32], the corrected estimator of genetic differentiation $\Phi_{ST}$ (Michalakis and Excoffier 1996) was used for multi-allelic markers, such as *MUM2* alleles and microsatellites. The corrected estimator $\Phi_{ST}$ takes into account the distances between alleles and has been shown to be the best estimator of differentiation for multi-allelic markers or haplotypes [32]. Differentiation was calculated between populations ($F_{ST}$) or between regions ($F_{CT}$), in a hierarchical setting taking into account the partition of genetic variation between populations within regions using the R package "hierfstat" (Goudet, 2005). Levels of differentiation for mucilage and *MUM2* fell within the range of differentiation observed at neutral markers (Table S3).

Goudet J (2005) hierfstat, a package for R to compute and test hierarchical F-statistics. Mol Ecol Notes 5: 184–186.

Michalakis Y, Excoffier L (1996) A generic estimation of population subdivision using distances between alleles with special reference for microsatellite loci. Genetics 142: 1061–1064.
