# Supplementary material for: Local Evolution of Seed Flotation in Arabidopsis
Source: PLoS Genet. 2014 Mar 13;10(3):e1004221. doi: 10.1371/journal.pgen.1004221 (PMC3953066; doi:10.1371/journal.pgen.1004221)
Supplement: Protocol S2 — Germination assay. (PDF) [file pgen.1004221.s007.pdf]

Protocol S2 Germination assay

Freshly harvested mature seeds were sown in triplicate in Petri dishes containing filter paper saturated with an aqueous solution of PEG 8000 at 10 to 20% (w/v) or a water control. Seeds were stratified at 4°C for 3 days before transfer to a growth chamber (50 μmol m^{-2} sec^{-1}, 16 h photoperiod, 25°C, 70% relative humidity). Germination was scored after four days based on the development of green cotyledons.
