# Supplementary material for: Local Evolution of Seed Flotation in Arabidopsis
Source: PLoS Genet. 2014 Mar 13;10(3):e1004221. doi: 10.1371/journal.pgen.1004221 (PMC3953066; doi:10.1371/journal.pgen.1004221)
Supplement: Protocol S3 — Water loss assays and Infrared thermography. (PDF) [file pgen.1004221.s008.pdf]

Protocol S3 Water loss assays and Infrared thermography

Rapid dehydration assays were carried out using three-week-old plants grown in compost (Tref Substrates) in the glasshouse (18-28°C minimum 13 h photoperiod). Four rosettes per genotype were cut from the root system and water loss measured as previously described (North et al., 2005). Resistance to progressive drought stress was determined as described by Plessis et al. [57] using eight plants per genotype and withholding water from four plants after three weeks. Infrared thermography was carried out as described previously [57].

North HM, Frey A, Boutin J-P, Sotta B, Marion-Poll A (2005) Analysis of xanthophyll cycle gene expression during the adaptation of Arabidopsis to excess light and drought stress : changes in RNA steady-state levels do not contribute to short-term responses. Plant Sci 169: 115-124.
