# Supplementary material for: Local Evolution of Seed Flotation in Arabidopsis
Source: PLoS Genet. 2014 Mar 13;10(3):e1004221. doi: 10.1371/journal.pgen.1004221 (PMC3953066; doi:10.1371/journal.pgen.1004221)
Supplement: Protocol S4 — Measurement of dry seed size and weight. (PDF) [file pgen.1004221.s009.pdf]

Protocol S4 Measurement of dry seed size and weight

Seed size was measured from images of seeds using Image J 1.43r (Freeware, National Institutes of Health, USA, http://rsb.info.nih.gov/ij/). Batches of 50 seeds were prepared with the Elmor C3 counting machine (Elmor, http://www.elmor.com/) then weighed on a Sartorius M2P microbalance (Sartorius, http://www.sartorius.com/).
