# Supplementary material for: Local Evolution of Seed Flotation in Arabidopsis
Source: PLoS Genet. 2014 Mar 13;10(3):e1004221. doi: 10.1371/journal.pgen.1004221 (PMC3953066; doi:10.1371/journal.pgen.1004221)
Supplement: Table S1 — Individuals characterized in detail from central Asian and Scandinavian populations affected in mucilage release. (PDF) [file pgen.1004221.s010.pdf]

Table S1 Individuals characterized in detail from central Asian and Scandinavian populations affected in mucilage release

Accession	Stock number/ donor	Country of origin	Site of origin	Latitude (N)	Longitude (E)
Ale-8	618AV/Ågren	Sweden	Sandy soil in clearing of open pine forest	56.42	16.31
Dja-1	534AV/Loudet	Kyrgyzstan	Rocky slope	42.589767	73.629717
Had-3-1	625AV/Rognli	Norway	South abrupt rocks in small dry patches of cultivated landscape and sheep pasture	68.507617	14.89235
Lom-3-1	624AV/Rognli	Norway	Sheepwalk in cultivated landscape, dry ground	61.681333	8.231000
Neo-3	539AV/Loudet	Tadjikistan	Flat rocky meadow	37.357333	72.467867
Neo-6	540AV/Loudet	Tadjikistan	Flat rocky meadow	37.357333	72.467867
Nfro-1-1	622AV/Rognli	Norway	SW slope of mountain pasture landscape	61.57745	9.661783
Sk-1-1	623AV/Rognli	Norway	By old cart road on SW soil cliff in old cultivated landscape	61.898967	8.253983
Sku-20	621AV/Ågren	Sweden	Rocky ledge of steep SE facing mountain slope	63.05	18.22
Sus-1	533AV/Loudet	Kyrgyzstan	River bank	42.188033	73.406933

Coordinates are given in decimal degree format. AV numbers correspond to Versailles Arabidopsis stock centre.
