# Supplementary material for: Local Evolution of Seed Flotation in Arabidopsis
Source: PLoS Genet. 2014 Mar 13;10(3):e1004221. doi: 10.1371/journal.pgen.1004221 (PMC3953066; doi:10.1371/journal.pgen.1004221)
Supplement: Table S2 — Polymorphisms present in the MUM2 gene between accessions affected in mucilage release. (PDF) [file pgen.1004221.s011.pdf]

Table S2 Polymorphisms present in the *MUM2* gene between accessions affected in mucilage release

Position (in bp from Col ATG)	**102**	**165**	406/407	409	549/550	616	674	693	718	729-733	740	758	943
	Exon 1	Exon 1	Intron 1	Intron 1	Intron 1	Intron 1	Intron 1	Intron 1	Intron 1	Intron 1	Intron 1	Intron 1	Intron 1
Col-0	C	T	CG	a	ct	t	a	g	g	gtttt	g	g	a
Bay-0	T	T	CG	t	ct	t	a	g	t	---tt	g	a	a
Shahdara / Neo-3	C	T	CG	a	--	t	a	g	t	---tt	g	g	a
Neo-6	C	T	CG	a	--	t	a	g	t	---tt	g	g	a
Dja-1	C	T	CG	a	--	t	a	g	t	---tt	g	g	a
Sus-1	C	T	CG	a	ct	t	a	g	t	ttttt	a	g	a
Nfro-1-1	C	T	CG	a	ct	c	a	g	t	ttttt	g	g	a
Ale-8	C	C	TT	a	ct	t	a	a	t	-----	g	g	g
Sku-20	C	C	TT	a	ct	t	a	a	t	-----	g	g	g
Had-3-1	C	T	CG	a	ct	t	a	a	t	---tt	g	g	g
Lom-3-1	C	T	CG	a	ct	t	-	g	t	---tt	g	g	a
Sk-1-1	C	T	CG	a	ct	c	a	g	t	--ttt	g	g	a

Accession	945	962	1053	1066	1089	1203	1208	1256	1264	1307	1408	1575	1765	1846	1942/1943
	Intron 1	Intron 1	Intron 1	Intron 1	Intron 1	Intron 1	Intron 1	Intron 1	Intron 1	Intron 1	Intron 1	Intron 1	Intron 1	Intron 1	Intron 1
Col-0	a	c	a	t	a	g	g	c	c	a	a	c	g	g	tc
Bay-0	a	c	a	t	g	g	g	c	c	g	a	c	g	g	ct
Shahdara / Neo-3	a	c	a	t	g	t	g	c	c	g	t	c	g	g	ct
Neo-6	a	c	a	t	g	g	g	c	c	g	t	c	g	g	ct
Dja-1	a	c	a	t	g	g	g	c	c	g	t	c	g	g	ct
Sus-1	a	t	a	t	g	g	g	c	c	g	a	c	c	g	ct
Nfro-1-1	a	c	a	t	g	g	g	c	c	g	a	c	g	g	tc
Ale-8	g	t	g	t	a	g	g	c	t	g	a	t	g	a	ct
Sku-20	g	t	g	c	a	g	g	c	t	g	a	t	g	g	ct
Had-3-1	g	c	g	c	a	g	a	a	t	g	a	t	g	a	ct
Lom-3-1	a	c	a	t	g	g	g	c	c	a	a	c	g	g	tc
Sk-1-1	a	c	a	t	g	g	g	c	c	g	a	c	g	g	tc

Accession	1985[]	1996[]	2010	2189	2198	**2323**	2406	2461	**2540**	**2580**	2695[]	2700	**2817**	3122	3134
	Intron 1	Intron 1	Intron 1	Intron 2	Intron 2	Exon 3	Intron 3	Intron 3	Exon 4	Exon 4	Intron 4	Intron 4	Exon 5	Intron 6	Intron 6
Col-0	-----	----	c	c	t	C	t	c	G	G	---------------	t	G	t	a
Bay-0	gatat	ccaa	c	a	t	C	c	c	G	G	---------------	t	G	t	a
Shahdara / Neo-3	gatat	ccaa	c	a	t	C	t	c	G	G	ccgaaccaaa	t	G	a	g
Neo-6	gatat	ccaa	c	a	t	T	t	c	G	G	ccgaaccaaa	t	G	a	g
Dja-1	gatat	ccaa	c	a	t	C	t	c	C	G	ccgaaccaaa	t	G	a	g
Sus-1	gatat	ccaa	g	a	t	C	t	c	G	A	---------------	c	A	a	a
Nfro-1-1	-----	----	c	c	t	C	t	t	G	G	---------------	t	G	a	a
Ale-8	-----	----	c	a	a	C	t	c	G	G	---------------	t	G	a	a
Sku-20	-----	----	c	a	a	C	t	c	G	G	---------------	t	G	a	a
Had-3-1	-----	----	c	a	a	C	t	c	G	G	---------------	t	G	a	a
Lom-3-1	-----	----	c	c	t	C	t	t	G	G	---------------	t	G	a	a
Sk-1-1	-----	----	c	c	t	C	t	t	G	G	---------------	t	G	a	a
						=> STOP			M => I	E => K			=> STOP		

Accession	**3165**	**3201**	**3425**	4253	4393	**4421**	**4840**	**4845**	**4906[]**	4966	**5029**	**5102-5145**	**5110**
	Exon 7	Exon 7	Exon 8	Intron 11	Intron 11	Exon 12	Exon 14	Exon 14	Exon 14	Intron 14	Exon 15	Exon 15	Exon 15
Col-0	C	T	G	a	a	G	T	T	----	g	C	TCACCCCT	G
Bay-0	C	T	G	a	a	G	T	T	----	g	C	TCACCCCT	G
Shahdara / Neo-3	C	T	G	g	g	G	T	T	----	g	C	---------------	G
Neo-6	C	T	G	g	g	G	T	T	----	g	C	TCACCCCT	G
Dja-1	C	T	G	g	g	G	T	T	----	g	C	TCACCCCT	G
Sus-1	C	T	G	a	a	G	G	G	----	a	A	TCACCCCT	T
Nfro-1-1	G	T	G	g	a	G	T	T	----	g	C	TCACCCCT	G
Ale-8	C	T	T	a	a	G	T	T	----	g	A	TCACCCCT	G
Sku-20	C	T	T	a	a	G	T	T	----	g	A	TCACCCCT	G
Had-3-1	C	C	G	a	a	A	T	T	TACA	g	A	TCACCCCT	G
Lom-3-1	C	T	G	g	a	G	T	T	----	g	C	TCACCCCT	G
Sk-1-1	C	T	G	g	a	G	T	T	----	g	C	TCACCCCT	G
	F => L		=> STOP			G => E			=> frameshift + STOP			=> frameshift + STOP	

Each column is a polymorphic site (either single nucleotide polymorphisms or insertions/deletions) in the *MUM2* gene (At5g63800). Each line is the haplotype of the accession indicated at the top left, except for the top two lines that indicate the position (in bp) for each polymorphic site from the ATG of the gene, or exon or intron number. Alleles identical to Col-0 are shaded in black, those in exons are shown in upper case and in introns are shown in lower case. Non-synonymous polymorphisms are highlighted in red and the consequences in terms of protein sequence are indicated below columns.
