# Supplementary material for: Local Evolution of Seed Flotation in Arabidopsis
Source: PLoS Genet. 2014 Mar 13;10(3):e1004221. doi: 10.1371/journal.pgen.1004221 (PMC3953066; doi:10.1371/journal.pgen.1004221)
Supplement: Table S3 — Analysis of geographical variation in Arabidopsis mucilage. (PDF) [file pgen.1004221.s012.pdf]

Table S3 Analysis of geographical variation in Arabidopsis mucilage

	**Within region variation**		**Between region variation**	
	**Asia**	**Norway**	**Asia and Norway**	**Asia, France, Norway, Spain**
Mucilage	F_{ST} = 1	F_{ST} = 0.85	F_{CT} = 0	F_{CT} = 0.123
MUM2	F_{ST} = 0.84	F_{ST} = 0.86	F_{CT} = 0.41	not analyzed
Neutral markers *	F_{ST} = 0 – 1	F_{ST} = 0 – 1	F_{CT} = 0 – 0.90	F_{CT} = 0.06-0.24

SNP, single nucleotide polymorphism; F_{ST}, fixation index among populations; F_{CT}, fixation index among regions. For neutral markers, the 95 percentiles of the distribution observed for 137 SNP markers and 20 microsatellite markers is given. Values observed for mucilage and *MUM2* fall within the 95 percentile of the neutral markers.
