# Supplementary material for: Local Evolution of Seed Flotation in Arabidopsis
Source: PLoS Genet. 2014 Mar 13;10(3):e1004221. doi: 10.1371/journal.pgen.1004221 (PMC3953066; doi:10.1371/journal.pgen.1004221)
Supplement: Table S4 — Central Asian and Scandinavian accessions analysed for seed flotation. (PDF) [file pgen.1004221.s013.pdf]

Table S4. Central Asian and Scandinavian accessions analyzed for seed flotation

Accession	Versailles identification number (AV)	Country of origin	Latitude (N)	Longitude (E)
9481B	261	Kazakhstan	-	-
Al-0	456	Denmark	-	-
Bas-1	347	Russia	51.8333	79.4666
Ber	299	Denmark	-	-
Bij	355	Russia	52.5166	85.2666
Chab-1	351	Russia	53.6	79.3833
Chi-0	89	Russia	-	-
Fl-1	199	Finland	-	-
Fl-3	472	Finland	-	-
Hau-0	273	Denmark	-	-
Hodja-Obi-Garm	203	Tadjikistan	-	-
K-oz-1	333	Russia	51.3333	82.1833
K-oz-3	335	Russia	51.3333	82.1833
Kly-2	328	Russia	51.3	82.5833
Kly-4	330	Russia	51.3166	82.5666
Kly-5	331	Russia	51.3333	82.55
Kondara	190	Tadjikistan	-	-
Kz-1	402	Kazakhstan	49.5	73.1
Kz-9	403	Kazakhstan	49.5	73.1
* Leb-1	336	Russia	51.6333	80.7833
Leb-4	339	Russia	51.6333	80.8
Lu-1	54	Sweden	-	-
Mas	354	Russia	54.1166	81.3
N3	291	Russia	-	-
N6	262	Russia	-	-
N7	263	Russia	-	-
N8	264	Russia	-	-
N9	284	Russia	-	-
N10	265	Russia	-	-
N11	285	Russia	-	-
N13	266	Russia	-	-
N15	268	Russia	-	-
N16	287	Russia	-	-
N17	269	Russia	-	-
Nos	343	Russia	51.8666	80.5833
Nov-3	342	Russia	51.7333	80.85
Oy-0	224	Norway	-	-
Pan	353	Russia	53.8166	80.3
Per-1	100	Russia	-	-
* Rak-1	344	Russia	51.85	80.05
* Rak-3	346	Russia	51.8666	80.05
Rld-2	229	Russia	-	-
Rsch-4	90	Russia	-	-
Rubezhnoe-1	231	Ukraine	-	-
* Sev	350	Russia	52.1	79.3
Sij-1	356	Uzbekistan	41.75	70.0833
Sij-4	359	Uzbekistan	41.75	70.0833
St-0	62	Sweden	-	-
Stw-0	92	Russia	-	-
Sv-0	513	Denmark	-	-
Te-0	68	Finland	-	-
Ull2-3	383	Sweden	56.09	13.46
Wil-3	525	Russia	-	-

Co-ordinates are given in decimal degree format. -, precise location data unavailable.

*, indicates floating mucilage releasing (FMR) accessions.
